# Structural and energetic insights into Mn-to-Fe substitution in the oxygen-evolving complex

**DOI:** 10.1016/j.isci.2023.107352

**Published:** 2023-07-08

**Authors:** Masahiro Saito, Keisuke Saito, Hiroshi Ishikita

**Affiliations:** 1 Department of Applied Chemistry, The University of Tokyo, 7-3-1 Hongo, Bunkyo-ku, Tokyo 113-8654, Japan; 2 Research Center for Advanced Science and Technology, The University of Tokyo, 4-6-1 Komaba, Meguro-ku, Tokyo 153-8904, Japan

**Keywords:** Chemistry, Catalysis, Photoabsorption

## Abstract

Manganese (Mn) serves as the catalytic center for water splitting in photosystem II (PSII), despite the abundance of iron (Fe) on earth. As a first step toward why Mn and not Fe is employed by Nature in the water oxidation catalyst, we investigated the Fe_4_CaO_5_ cluster in the PSII protein environment using a quantum mechanical/molecular mechanical (QM/MM) approach, assuming an equivalence between Mn(III/IV) and Fe(II/III). Substituting Mn with Fe resulted in the protonation of *μ*-oxo bridges at sites O2 and O3 by Arg357 and D1-His337, respectively. While the Mn_4_CaO_5_ cluster exhibits distinct open- and closed-cubane S_2_ conformations, the Fe_4_CaO_5_ cluster lacks this variability due to an equal spin distribution over sites Fe1 and Fe4. The absence of a low-barrier H-bond between a ligand water molecule (W1) and D1-Asp61 in the Fe_4_CaO_5_ cluster may underlie its incapability for ligand water deprotonation, highlighting the relevance of Mn in natural water splitting.

## Highlights

• Substituting Mn with Fe results in the protonation of specific O sites in Fe_4_CaO_5_
• Fe_4_CaO_5_ lacks the open- and closed-cubane S_2_ conformations observed in Mn_4_CaO_5_
• O6 incorporation during the S_2_ to S_3_ transition is unlikely in Fe_4_CaO_5_
• The release of the proton from a ligand water molecule is inhibited in Fe_4_CaO_5_

## Graphical abstract

**Figure:**
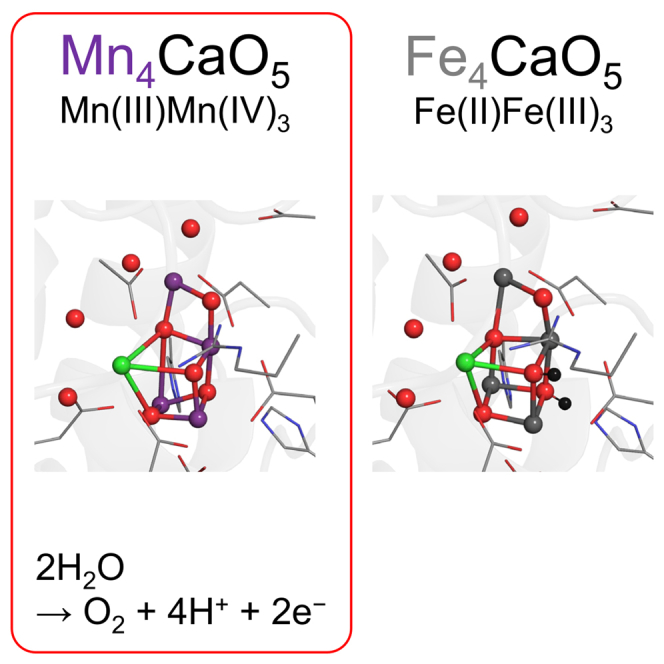

## Introduction

O_2_ evolution occurs at the catalytic center, the Mn_4_CaO_5_ cluster in photosystem II (PSII).^1^ The Mn_4_CaO_5_ cluster comprises the Mn_3_CaO_4_ cubane region (Mn1, Mn2, Mn3, Ca^2+^, O1, O2, O3, and O5) and the dangling region (Mn4 and O4).^2^ The most stable oxidation state, S_1_, is Mn(III)_2_Mn(IV)_2_ in the high oxidation state model (corresponding to S_3_ in the low oxidation model^3^). Light-induced electron transfer to redox-active D1-Tyr161 (TyrZ) increases the oxidation state of the Mn_4_CaO_5_ cluster from S_1_ via S_2_ and S_3_ to S_0_. The release of the proton occurs with a stoichiometry of 1:0:1:2 for the S_0_ to S_1_, S_1_ to S_2_, S_2_ to S_3_, and S_3_ to S_0_ transitions. O_2_ evolves during the S_3_ to S_0_ transition.^4^ The release of the proton occurs along the O4-water chain in the S_0_ to S_1_ transition,^5^^,^^6^^,^^7^ whereas it occurs via D1-Asp61 along the D1-Glu65/D2-Glu312 channel in the S_2_ to S_3_ transition.^8^^,^^9^ D1-Asp61 forms an H-bond with a ligand water molecule at the dangling Mn4 site, W1.^2^

S_2_ is a characteristic intermediate state, as it proceeds to the S_2_ to S_3_ transition via proton-coupled electron transfer^7^^,^^8^^,^^10^^,^^11^^,^^12^^,^^13^^,^^14^^,^^15^^,^^16^^,^^17^ and substrate-water incorporation.^18^^,^^19^^,^^20^^,^^21^^,^^22^ Electron spin echo envelope modulation (ESEEM) and electron nuclear double resonance (ENDOR) studies have suggested that all of the *μ*-oxo bridges of the Mn_4_CaO_5_ cluster are deprotonated in S_2_ (i.e., Mn(III)Mn(IV)_3_).^23^^,^^24^ Theoretical studies suggest that S_2_ has two distinct conformations, the open- and closed-cubane conformations with (Mn1, Mn2, Mn3, Mn4) = (III, IV, IV, IV) and (IV, IV, IV, III), respectively. The open-cubane S_2_ conformation was identified in the X-ray free-electron laser (XFEL) structures, but not the closed-cubane S_2_ conformation,^18^^,^^19^^,^^20^^,^^21^^,^^22^ which may be due to the open-cubane S_2_ conformation being energetically more stable than the closed-cubane S_2_ conformation.^25^^,^^26^^,^^27^^,^^28^ Recent theoretical studies performed in the presence of the PSII protein environment suggested that the *g* = 4.1 signal observed for plant PSII in electron paramagnetic resonance (EPR) spectroscopy^29^^,^^30^ corresponds to the closed-cubane S_2_ conformation with W1 = OH^–^.^31^ Mn1(III) … O5 is long and Mn4(IV) … O5 is short in the open-cubane S_2_ conformation, whereas Mn1(IV) … O5 is short and Mn4(III) … O5 is long in the closed-cubane S_2_ conformation.^32^^,^^33^ Theoretical studies proposed that the substrate water molecule could be incorporated into the O5 moiety.^10^^,^^11^^,^^12^^,^^14^^,^^15^^,^^16^ According to the XFEL structures, a water molecule is incorporated into the O5 moiety during the S_2_ to S_3_ transition.^18^^,^^19^^,^^20^^,^^21^^,^^22^

Notably, Cl^−^ and Ca^2+^ are required to proceed the S_2_ to S_3_ transition. The PSII crystal structure shows that a chloride ion, Cl-1, is located at D1-Asn181 and D2-Lys317.^2^ When Cl^−^ is depleted, the S-state transition is inhibited at the S_2_TyrZ^⋅^ formation.^34^ Indeed, electron transfer from S_2_/S_3_ to TyrZ is energetically uphill due to an increase in the S_2_/S_3_ redox potential in Cl^−^-depleted PSII.^35^ A salt bridge forms between D1-Asp61 and D2-Lys317 upon the depletion of Cl^–^,^36^^,^^37^ which may also inhibit proton transfer from W1 via D1-Asp61 toward the bulk region. The salt bridge and Cl^−^ (Cl-1) are absent in D2-Lys317Ala PSII. Nevertheless, electron transfer can occur during the S_2_ to S_3_ transition even in the absence of Cl-1,^37^ which suggests that Cl-1 is not necessarily required for the electron transfer process in the S_2_ to S_3_ transition.

The inhibition of the S_2_ to S_3_ transition in Cl^−^-depleted PSII resembles that in Ca^2+^-depleted PSII.^38^^,^^39^^,^^40^^,^^41^ Ca^2+^ depletion not only causes the alteration of the H-bond network at the Mn_4_O_5_ and TyrZ moieties^26^ but also decreases the redox potential of TyrZ significantly due to reorientation of the water molecules in the H-bond network, making electron transfer from the Mn_4_CaO_5_ cluster to TyrZ uphill.^42^ Replacement of Ca^2+^ with any metals except Sr^2+^ inhibits O_2_ evolution.^38^^,^^43^^,^^44^^,^^45^^,^^46^ The geometry of the catalytic site in Sr^2+^-substituted PSII (Sr^2+^-PSII) resembles that of native PSII (Ca^2+^-PSII).^47^^,^^48^ The *E*_m_ values for the artificial clusters with Sr^2+^ are also similar to those with Ca^2+^.^49^^,^^50^^,^^51^ Thus, Ca^2+^ is a prerequisite for the O_2_ evolving activity in native PSII but can functionally be substituted with Sr^2+^.^52^

In contrast, Mn is indispensable for the light-driven catalytic activity of the Mn_4_CaO_5_ cluster in the PSII protein environment. In the artificial cluster with the Fe_5_O core, electrocatalytic O_2_ evolution was reported.^53^ However, no O_2_ evolution was observed in the PSII membrane when two of the four Mn sites were substituted with Fe.^54^ Understanding why Mn is chosen over Fe as the redox-active metal in the PSII catalytic center raises several fundamental questions: Why did Nature pick Mn and not Fe for the water-oxidation catalyst? Would there be any advantageous properties in the electronic state of Mn compared to Fe in the context of the PSII catalytic center? What would be the protonation states of the bridges and ligands, considering the evolutionary optimization for higher valence of Mn(III/IV)? What would be the activation energies for S state transitions? Would Fe also exhibit two characteristic conformations, corresponding to the open- and closed-S_2_ conformations observed in the Mn_4_CaO_5_ cluster? What would the active site look like if it were optimized by evolution to operate with Fe instead of Mn? Is there a chance that it would function effectively in that scenario? Some of these questions were put forwarded by Armstrong previously.^52^

To the best of our knowledge, the detailed characterization of the Fe_4_CaO_5_ cluster in the PSII protein environment has not been reported. Investigating the properties of the Fe_4_CaO_5_ cluster in the PSII protein environment could provide valuable insights into the reasons behind the selection of Mn instead of the more abundant Fe^55^ for the catalytic center. Here, we investigate the structural and energetic details of the Fe_4_CaO_5_ cluster in the characteristic intermediate state, S_2_, using a quantum mechanical/molecular mechanical (QM/MM) approach, considering the entire PSII protein environment (i.e., all protein subunits and cofactors), and assuming that Mn(III/IV) corresponds to Fe(II/III). During QM/MM calculations, the decrease in the cluster net charge caused by substitution of Mn with Fe can be compensated for by the release of protons from titratable groups,^56^ including adjacent water molecules,^57^ if energetically favorable. The protonation states and oxidation states of the Fe_4_CaO_5_ cluster are currently unknown, unlike the extensively studied Mn_4_CaO_5_ cluster. By investigating these protonation states and oxidation states, our aim is to gain a deeper understanding of the unique characteristics and potential differences between Mn and Fe in the context of the water-oxidizing catalyst. Additionally, the analysis of the resulting protonation state of the Fe_4_CaO_5_ cluster may provide insights into why only two Mn sites were replaced with Fe in the Ca^2+^-depleted PSII.^54^

## Results and discussion

### Protonation state of the Fe_4_CaO_5_ cluster

To appropriately model and define the Fe_4_CaO_5_ cluster, the presumed oxidation and protonation states of the Fe_4_CaO_5_ cluster must be considered. While the Mn_4_CaO_5_ cluster utilizes two typical valence states, Mn(III) and Mn(IV), it is assumed that the Fe_4_CaO_5_ cluster employs Fe(II) and Fe(III).

The stable oxidation and protonation states of the Fe_4_CaO_5_ cluster remain unknown. In most studies on the Mn_4_CaO_5_ cluster, the lower S-states, namely, S_0_, S_1_, and S_2_, are defined based on the number of oxidized Mn ions (Mn(IV)), i.e., Mn(III)_3_Mn(IV) in S_0_, Mn(III)_2_Mn(IV)_2_ in S_1_, and Mn(III)Mn(IV)_3_ in S_2_. Following this analogy, the corresponding S-states in the Fe_4_CaO_5_ cluster are tentatively considered as Fe(II)_3_Fe(III) in S_0_, Fe(II)_2_Fe(III)_2_ in S_1_, and Fe(II)Fe(III)_3_ in S_2_. While ESEEM and ENDOR studies have suggested the deprotonation of all *μ*-oxo bridges of the Mn_4_CaO_5_ cluster in S_2_ (i.e., Mn(III)Mn(IV)_3_),^23^^,^^24^ these protonation states may not necessarily hold true for the corresponding S-states of the Fe_4_CaO_5_ cluster due to the difference in the net charge between Mn_4_ and Fe_4_.

**Figure 1:**
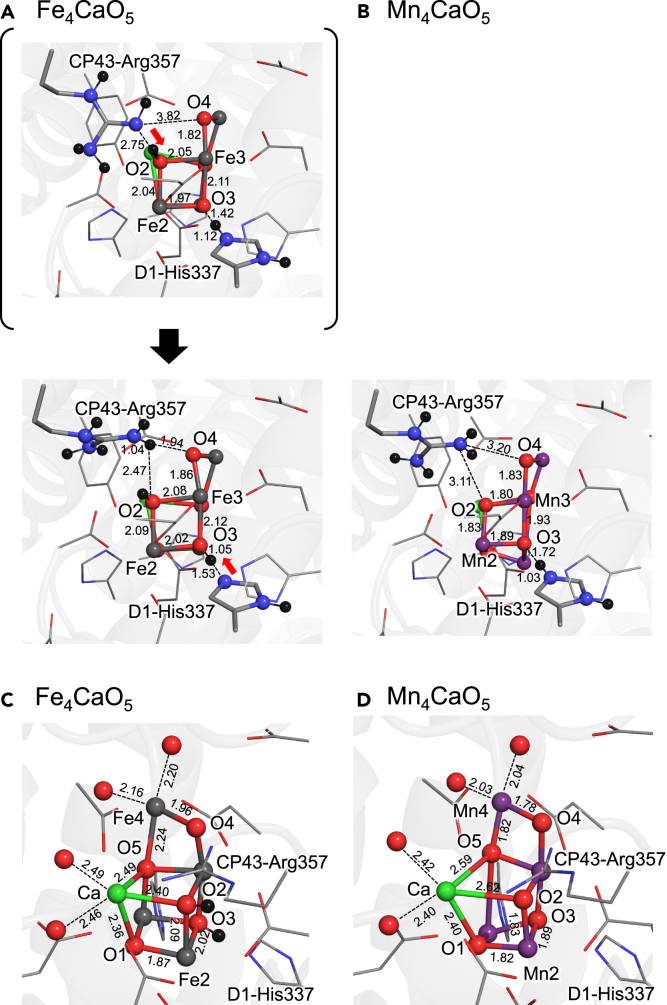
Protonation state of CP43-Arg357 and D1-His337 and overview of the clusters (A) Fe_4_CaO_5_ cluster in Fe(II)Fe(III)_3_. (B) Mn_4_CaO_5_ cluster in Mn(III)Mn(IV)_4_ (open-cubane S_2_ conformation). (C) Overview of the Fe_4_CaO_5_ cluster. (D) Overview of the Mn_4_CaO_5_ cluster. Only H atoms of CP43-Arg357 and D1-His337 are explicitly shown (black ball) for clarity. The black thick arrow indicates the protonation event. Red arrows indicate movement of protons during QM/MM calculations.

When all of the *μ*-oxo bridges of the Fe_4_CaO_5_ cluster are deprotonated in the corresponding Fe(II)Fe(III)_3_ state, CP43-Arg357 and D1-His337 release the protons toward O2 and O4, respectively (Figure 1A, top). Consequently, the Fe_4_CaO_5_ cluster is doubly protonated with OH^−^ at O2 and O3 in S_2_, which partially compensates for the loss of the positive charge of Fe(II/III) with respect to Mn(III/IV) (Figure 1B). The corresponding protonation events were also reported for the overreduced Mn_4_CaO_5_ cluster.^57^ Thus, the Fe_4_CaO_5_ cluster may resemble the overreduced Mn_4_CaO_5_ cluster.

Deprotonated CP43-Arg357 is likely to eventually reprotonate in equilibrium, as the p*K*_a_ value of CP43-Arg357 is sufficiently high even in Mn-PSII.^58^ In the Mn_4_CaO_5_ cluster, the release of the proton preferentially occurs from the *μ*-oxo bridges of the cluster (e.g., O4 in the S_0_ to S_1_ transition^5^^,^^6^^,^^7^) with respect to the ligand water molecule (e.g., W1 in the S_2_ to S_3_ transition^8^^,^^9^^,^^59^^,^^60^^,^^61^). The presence of the protonated O2 site in the Fe_4_CaO_5_ cluster suggests that the release of the proton from the ligand water molecule cannot proceed even in S_2_. Thus, the protonation of the Fe_4_CaO_5_ cluster is disadvantageous for water oxidation even if the loss of the net charge of the cluster is partially compensated for by the proton.

In contrast, D1-His337 cannot reorient due to the presence of D1-Glu333 (Nδ_D1-His337_ … O_D1-Glu333_ = 3.4 Å^2^). In addition, O3 shares the proton with D1-His337 via a low-barrier H-bond in lower S states of Mn-PSII^57^^,^^62^ and the low-dose structure.^56^^,^^63^ Thus, D1-His337 is charge-neutral, singly protonated in the presence of OH^−^ at O3 in Fe-PSII. QM/MM calculations suggest that protonated CP43-Arg357 is stable in the presence of OH^−^ at O2 and O3 in Fe-PSII, forming an H-bond with another H-bond acceptor, O4 (Figure 1A, bottom).

The cluster, in which two of the four Mn sites were substituted with Fe,^54^ may have similar characteristics of protonation state as the doubly protonated Fe_4_CaO_5_ cluster presented here (Figure 1) probably because of maintaining the charge balance. Below, the doubly protonated Fe_4_CaO_5_ cluster is investigated in the presence of singly protonated D1-His337 and protonated CP43-Arg357 in S_2_.

### Effect of metal substitution on molecular symmetry

Although the average angles formed by the Fe, Ca, and O sites in the Fe_4_CaO_5_ cluster and the corresponding angles in the Mn_4_CaO_5_ cluster are the same, there is a notable difference in the angle distribution. The Fe_4_CaO_5_ cluster exhibits a narrower angle distribution (89.3 ± 9.2°) than the Mn_4_CaO_5_ cluster (89.9 ± 14°) (Figure S1). This narrower angle distribution contributes to the Fe_4_CaO_5_ cluster appearing more cubic and symmetric in its molecular structure when compared to the Mn_4_CaO_5_ cluster (Figure 1). The contrasting angle distributions between the Fe and Mn clusters highlight the distinct molecular symmetries resulting from metal substitution.

### Absence of the open- and closed-cubane S_2_ conformations in the Fe_4_CaO_5_ cluster

In the Mn_4_CaO_5_ cluster, either Mn1 (closed-cubane conformation) or Mn4 (open-cubane conformation) is the reduced Mn(III) site in S_2_, as indicated by the spin density (Table 1). In the Fe_4_CaO_5_ cluster, however, the spin density is at the same level (∼4) for all four Fe sites. In addition, the spin state is equally distributed over the entire cluster, including the five O sites (Table 1). The equal distribution of the spin state over several Fe sites may be in line with the cubic and symmetric Fe_4_CaO_5_ structure when compared to the Mn_4_CaO_5_ cluster (Figures 1 and S1). The equal distribution of the spin state suggests that the Fe sites cannot be fully oxidized due to partial oxidation of the O sites, which is disadvantageous for decreasing the p*K*_a_ values of the substrate water molecules at the Fe moieties.

**Table 1:** Spin density of each metal site of the Fe_4_CaO_5_ and Mn_4_CaO_5_ clusters in S_2_

Fe_4_CaO_5_		Mn_4_CaO_5_ (open-cubane)	
Fe1	4.0	Mn1	3.8
Fe2	4.1	Mn2	3.0
Fe3	4.1	Mn3	3.0
Fe4	3.8	Mn4	3.0
O1	0.6	O1	0.0
O2	0.2	O2	0.0
O3	0.4	O3	0.0
O4	0.5	O4	0.1
O5	0.6	O5	0.0
Ca	0.0	Ca	0.0

The spin density of (4, 3, 3, 3) for (Mn1, Mn2, Mn3, Mn4) corresponds to the spin state of (↑↑↑↑) and the valence state of (III, IV, IV, IV), i.e., the open-cubane S_2_ conformation.

The spin exchange coupling between the two transition metals increases as the distance between the two metals decreases.^64^ The Fe … Fe distances in the Fe_4_CaS_5_ cluster are longer than those in the Fe_4_CaO_5_ cluster, as the Fe … S distances are longer than the Fe … O distances (Table S1). However, the spin state is more equally distributed over the five S sites in the Fe_4_CaS_5_ cluster than over the five O sites in the Fe_4_CaO_5_ cluster (Table S2). Thus, the equally distributed spin state in the Fe_4_CaO_5_ cluster cannot be explained by the short Fe … Fe distances.

The equal distribution of the spin state over several Fe sites is also observed in Fe_4_S_4_ clusters F_A_ and F_B_ located at the typical ferredoxin-like binding motif (CxxCxxCxxxCP) in photosystem I, in which a mixed valence Fe^2.5+^ … Fe^2.5+^ pair forms^65^^,^^66^ due to exchange coupling of the overlap of the d orbitals of Fe sites via the p orbital of bridging –S–.^64^ However, it should also be noted that equal distribution of the spin state over several Fe sites is not a characteristic of all Fe complexes. For example, a mixed valence Fe^2.5+^ … Fe^2.5+^ pair has not been reported for the Fe_4_S_4_ cluster F_X_ located on the preudo-*C*_2_ axis of the reaction center in photosystem I.^65^ As the size of the Fe_4_CaS_5_ cluster differs significantly from those of the Fe_4_CaO_5_ and Mn_4_CaO_5_ clusters (Table S1), below we focus on the Fe_4_CaO_5_ and Mn_4_CaO_5_ clusters (Figures 1C and 1D) if not otherwise specified.

**Figure 2:**
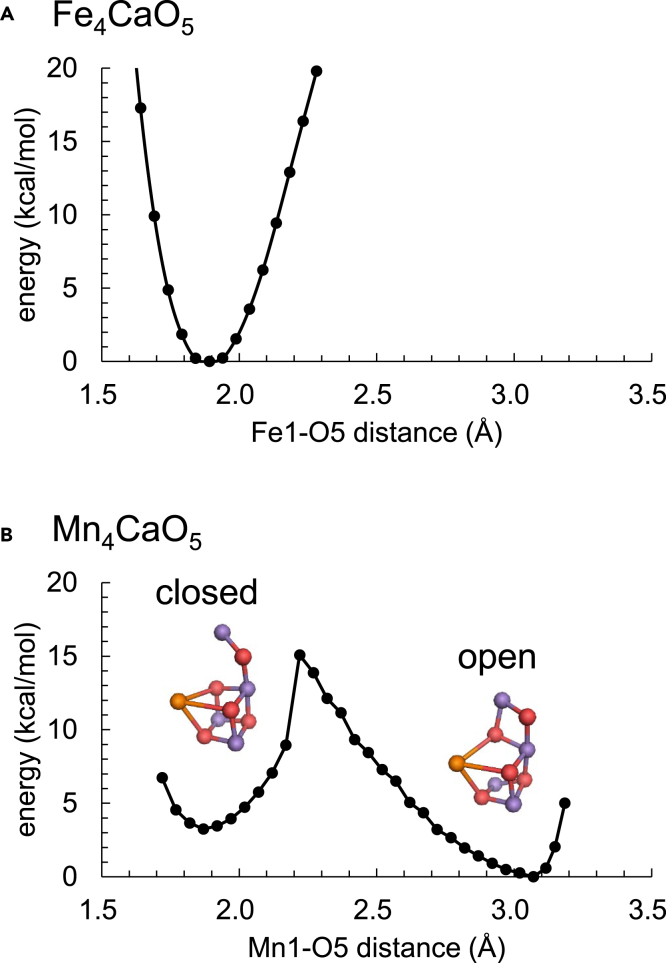
Potential energy profile for the O5 position along the Fe1 … O5 … Fe4 and Mn1 … O5 … Mn4 axes (A) Fe_4_CaO_5_ cluster. (B) Mn_4_CaO_5_ cluster. In the Mn_4_CaO_5_ cluster, the global energy minimum corresponds to the open-cubane S_2_ conformation, whereas the local energy minimum corresponds to the closed-cubane S_2_ conformation.

Intriguingly, the potential-energy profile for the O5 position along the Fe1 … Fe4 axis indicates that the open-cubane and closed-cubane S_2_ conformations do not exist in the Fe_4_CaO_5_ cluster in contrast to the Mn_4_CaO_5_ cluster (Figure 2). Instead, only a single S_2_ conformation, in which both Fe1 … O5 (1.89 Å) and O5 … Fe4 (2.24 Å) distances are short (Table S1), exists in the Fe_4_CaO_5_ cluster (Figures 1C and 2A). Two distinct conformations exist in the Mn_4_CaO_5_ cluster, because either Mn1 or Mn4 is the reduced Mn(III) site in S_2_. The absence of the distinct reduced Fe(II) site owing to equal distribution of the spin/valence states (Table 1) can explain the absence of the two corresponding conformations in the Fe_4_CaO_5_ cluster.

The XFEL structures of PSII imply that a water molecule is incorporated into the O5 moiety of the Mn_4_CaO_5_ cluster during the S_2_ to S_3_ transition.^18^^,^^19^^,^^20^^,^^21^^,^^22^ According to theoretical studies, the Mn1 … O5 moiety may serve as the O6 binding site in the open-cubane S_2_ conformation or the Mn4 … O5 moiety may serve as the O6 binding site in the closed-cubane S_2_ conformation (e.g.,^10^^,^^11^^,^^12^^,^^14^^,^^15^^,^^16^). The absence of the two S_2_ conformations suggests that O6 incorporation is unlikely to proceed in the Fe_4_CaO_5_ cluster during the S_2_ to S_3_ transition.

It should also be noted that Mn(III) has a low redox-potential value, as Mn(III) aqua species tend to undergo disproportionation to form Mn(II) and Mn(IV),^52^ while Fe(II) does not exhibit similar disproportionation behavior. Additionally, Fe(IV) is highly oxidizing, making it challenging to stabilize a Fe(IV) species in the S state.^52^ This difference in redox properties between Mn and Fe may also pose a disadvantage for the Fe_4_CaO_5_ cluster compared to the Mn_4_CaO_5_ cluster in terms of its stability and suitability for water oxidation.

### Absence of the release of the proton from W1 in the Fe_4_CaO_5_ cluster

**Figure 3:**
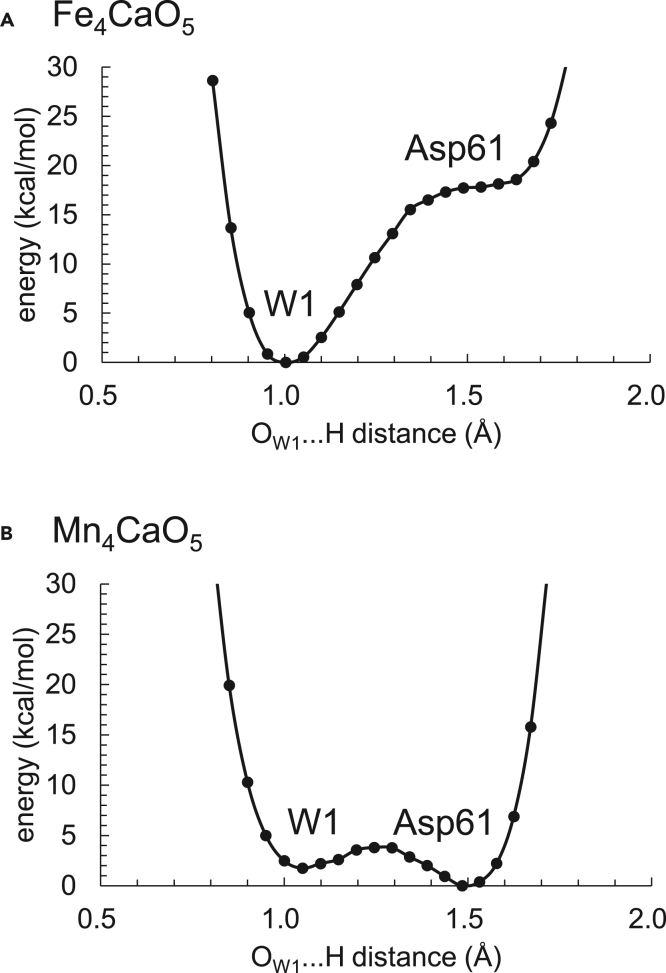
Potential energy profile for the H-bond between H_2_O at W1 and D1-Asp61 (A) Fe_4_CaO_5_ cluster. (B) Mn_4_CaO_5_ cluster. Labels W1 and Asp61 indicate the W1 and D1-Asp61 moieties in the H-bond, respectively.

W1 at the dangling Mn4(IV) site forms a low-barrier H-bond in the Mn_4_CaO_5_ cluster, facilitating the release of the proton from W1 via D1-Asp61 toward the lumenal protein surface^8^^,^^9^^,^^60^^,^^61^ (Figure 3B). However, the corresponding low-barrier H-bond does not form in the Fe_4_CaO_5_ cluster. The proton of H_2_O at W1 is localized at the W1 moiety, which suggests that proton transfer from W1 to D1-Asp61 is significantly inhibited in the Fe_4_CaO_5_ cluster with respect to in the Mn_4_CaO_5_ cluster (Figure 3A). It seems likely that the oxidation of the Fe sites does not provide a sufficient driving force for deprotonation of the ligand water molecule owing to the low valence, Fe(III), with respect to Mn(IV), inhibiting proton transfer.

### Changes in the H-bond network of TyrZ in the Fe_4_CaO_5_ cluster

**Figure 4:**
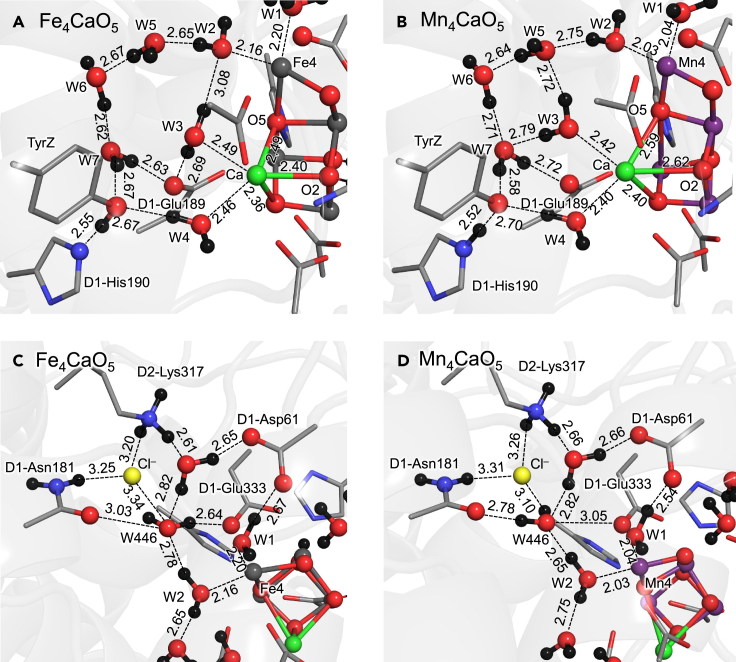
H-bond network of the Fe_4_CaO_5_ and Mn_4_CaO_5_ clusters (A) H-bond network of TyrZ for the Fe_4_CaO_5_ cluster. (B) H-bond network of TyrZ for the Mn_4_CaO_5_ cluster (open-cubane S_2_ conformation). (C) H-bond network near the Cl-1 binding site for the Fe_4_CaO_5_ cluster. (D) H-bond network near the Cl-1 binding site for the Mn_4_CaO_5_ cluster (open-cubane S_2_ conformation). Dotted lines indicate H-bonds.

W3 at Ca^2+^ donates H-bonds to W5 and W7, forming the diamond-shaped cluster of water molecules near the TyrZ … D1-His190 pair in the Mn_4_CaO_5_ cluster^67^ (Figure 4B). However, W3 donates H-bonds to W2 and D1-Glu189 in the Fe_4_CaO_5_ cluster, leading to the deformation of the diamond-shaped cluster of water molecules (Figure 4A). The observed deformation of the H-bond network due to the reorientation of W3 resembles the deformation of the H-bond network due to the reorientation of W3 in Mg^2+^-substituted PSII.^68^ The Fe_4_CaO_5_ cluster is slightly smaller than the Mn_4_CaO_5_ cluster, as the Ca^2+^ … O distances are shorter in the Fe_4_CaO_5_ cluster than in the Mn_4_CaO_5_ cluster (Table S1). The Mn_4_MgO_5_ cluster in Mg^2+^-PSII is slightly smaller than the Mn_4_CaO_5_ cluster in native PSII, as the Mg^2+^ radius is smaller than the Ca^2+^ radius.^68^ These characteristics suggest that the reorientation of W3 and the deformation of the H-bond network of TyrZ observed in the two clusters originate from the large cavity space with respect to the original Mn_4_CaO_5_ cluster.

**Figure 5:**
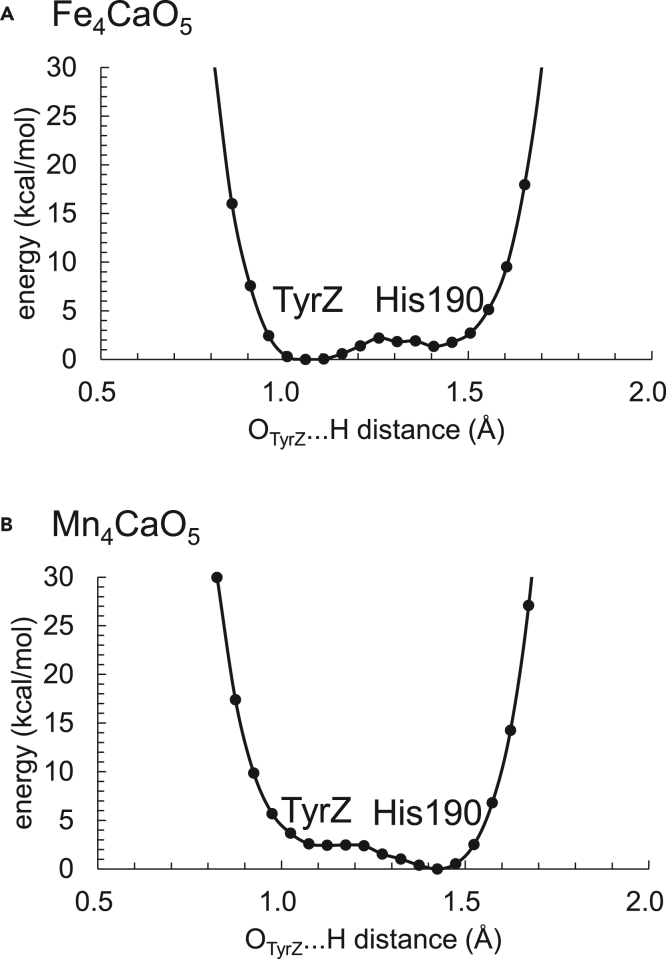
Potential energy profile for the H-bond between TyrZ and D1-His190 (A) Fe_4_CaO_5_ cluster. (B) Mn_4_CaO_5_ cluster. Labels TyrZ and His190 indicate the TyrZ and D1-His190 moieties in the H-bond, respectively. Although the shape of the potential-energy curve for the TyrZ … His190 of the Mn_4_CaO_5_ cluster (B) resembles that for the W1 … D1-Asp61 of the Mn_4_CaO_5_ cluster (Figure 3B) due to low-barrier H-bonds, the numerical data are different.

The TyrZ … D1-His190 distance is slightly longer in the Fe_4_CaO_5_ cluster (2.55 Å, Figure 4A) than in the Mn_4_CaO_5_ cluster (2.52 Å, Figure 4B). Nevertheless, the potential-energy profile for the H-bond suggests that the formation of a low-barrier H-bond between TyrZ and D1-His190 is not impaired even in the Fe_4_CaO_5_ cluster (Figure 5). Thus, the presence of low-barrier H-bonds cannot be judged by the H-bond distances but can be judged only by the shape of the potential-energy curve, as suggested by Schutz and Warshel.^69^ Although the redox potential of TyrZ is slightly affected by the S-state change,^70^^,^^71^^,^^72^ the H-bond characteristics of the TyrZ … D1-His190 pair is likely less sensitive to the charges of the Mn_4_CaO_5_ cluster, and the S-state changes than those of the other H-bonds (e.g., W1 … D1-Asp61, Figure 3). This may explain why the TyrZ … D1-His190 pair functions as an electron acceptor of the Mn_4_CaO_5_ cluster throughout the S-cycle.

### Chloride binding site in the Fe_4_CaO_5_ cluster

In contrast to the H-bond network of TyrZ, the H-bond network of the Cl^−^ binding site near the Mn_4_CaO_5_ cluster (Cl-1) remains essentially unchanged upon the substitution of Mn with Fe (Figures 4C and 4D). The slight increase in the Cl^−^ … O_W446_ distance (3.10 Å for the Mn_4_CaO_5_ cluster and 3.34 Å for the Fe_4_CaO_5_ cluster) is due to the increase in the cavity space, which in turn is compensated for by the decreases in the Cl^−^ … D1-Asn181 and Cl^−^ … D2-Lys317 distances. It seems likely that the binding affinity of Cl^−^ is at the same level in Mn-PSII and Fe-PSII.

It was reported that photoelectrochemical water oxidation in NaCl aqueous solution using most metal oxides, including Fe, suffers from the production of toxic HClO^−^ due to the oxidation of Cl^–^.^73^ HClO^−^ production was suppressed only when using Mn oxides.^73^ This is not the case for PSII. The present result indicates that the PSII protein environment does not allow Cl^−^ to approach even the Fe_4_CaO_5_ cluster (Figure 4C). D2-Lys317 not only serves as the binding site but also increases the redox potential for Cl^−/⋅^, inhibiting oxidation of Cl^−^ to HClO^−^. Instead, Cl^−^ is required to proceed with the S_2_ to S_3_ transition,^34^ i.e., ultimately O_2_ evolution. In the S_2_ to S_3_ transition, electron transfer from the Mn_4_CaO_5_ cluster to TyrZ is energetically uphill in the absence of Cl^–^.^35^ Furthermore, removal of Cl^−^ leads to the formation of a salt bridge between D1-Asp61 and D2-Lys317 in the proton transfer pathway, leading to inhibition of the release of the proton.^36^^,^^37^ Based on these observations and the findings presented in this study, it can be inferred that PSII likely opted for Mn(III/IV) rather than Fe(II/III) primarily to lower the p*K*_a_ values of the substrate water molecules (Figure 3). However, the redox potential value for Mn(III/IV) in the Mn_4_CaO_5_ cluster alone may not be sufficiently low to enable efficient electron transfer to TyrZ.^35^ To overcome this energetic challenge, PSII employs Cl^−^, which effectively decreases the redox potential. This strategic utilization of Cl^–^ ions allows for downhill electron transfer.^35^

### Conclusions

As the spin state is equally distributed over the entire Fe_4_CaO_5_ cluster in contrast to the Mn_4_CaO_5_ cluster (Table 1), the difference in the valence state is unclear between the Fe1 and Fe4 sites. Thus, the open-cubane and closed-cubane S_2_ conformations do not exist in the Fe_4_CaO_5_ cluster (Figure 2A). Instead, both Fe1 … O5 (1.89 Å) and O5 … Fe4 (2.24 Å) distances are too short (Table S1) to allow an external water molecule (e.g., O6^18^^,^^19^^,^^20^^,^^21^^,^^22^) to enter the O5 moiety in the S_2_ to S_3_ transition. The low-barrier H-bond between W1 and D1-Asp61, which is observed in the Mn_4_CaO_5_ cluster^8^^,^^60^^,^^61^ (Figure 3B), is absent in the Fe_4_CaO_5_ cluster (Figure 3A), inhibiting the release of the proton from W1 via D1-Asp61 toward the protein lumenal surface in the S_2_ to S_3_ transition.^9^ The H-bond network of TyrZ shows the difference specifically in the orientation of W3 at Ca^2+^ between the Fe_4_CaO_5_ and Mn_4_CaO_5_ clusters (Figures 4A and 4B). The deformation of the H-bond network does not affect the formation of the low-barrier H-bond between TyrZ and D1-His190 in the Fe_4_CaO_5_ cluster (Figure 5). The H-bond network of the Cl-1 binding site remains essentially unchanged upon the substitution of Mn with Fe, which suggests that harmful HClO^–^^73^ is unlikely to be generated in the Fe_4_CaO_5_ cluster as long as the PSII protein environment exists. Based on these observations, the Fe_4_CaO_5_ cluster may not function as a water-oxidation catalyst in the binding site of PSII primarily because it cannot support in this environment the efficient deprotonation of the substrate water molecules (Figure 3) or the incorporation of an external water molecule into the cluster (Figure 2), the events occurring during the S_2_ to S_3_ transition of the natural Mn_4_CaO_5_ catalyst.

### Limitations of the study

The results depend on the original atomic coordinates of the crystal structures. The original side-chain orientations may affect the results, although the geometries of the catalytic sites are quantum-chemically optimized.

## STAR★Methods

### Key resources table

**Table:** 

REAGENT or RESOURCE	SOURCE	IDENTIFIER
**Software and algorithms**		

CHARMM	Brooks et al.^74^	RRID:SCR_014892; https://www.charmm.org
QSite	QSite^75^	https://www.schrodinger.com/products/qsite
VMD	Humphrey et al.^76^	http://www.ks.uiuc.edu/Research/vmd/

### Resource availability

#### Lead contact

Further information and requests for resources should be directed to and will be fulfilled by the lead contact, Hiroshi Ishikita (hiro@appchem.t.u-tokyo.ac.jp).

#### Materials availability

This study did not generate new unique reagents.

### Method details

The atomic coordinates were obtained from the X-ray structure of PSII from *Thermosynechococcus vulcanus* (PDB code, 3ARC).^2^ H atoms were generated and energetically optimized using CHARMM,^74^ while heavy atoms were kept fixed and titratable groups were maintained in their standard protonation states (acidic groups ionized and basic groups protonated). D1-His337 was considered to be protonated.^77^ Atomic partial charges for amino acids and cofactors were obtained from the CHARMM22^78^ parameter set and previous studies.^5^

The QSite^75^ program was used, employing the unrestricted density functional theory method with the B3LYP functional and LANL2DZ∗ (i.e., LACVP∗) basis sets. 44 sodium ions were added as counter ions randomly around the protein to neutralize the entire system using the Autoionize plugin in VMD.^76^ In the QM region, all atom positions were relaxed (i.e., not fixed at the positions in the crystal structure). In the MM region, the H-atom positions were relaxed, and the heavy-atom positions were fixed using the OPLS2005 force field.^79^ The Fe_4_CaO_5_ cluster was considered to be in the S_2_-corresponding state, Fe(II)[Fe(III)]_3_, with ferromagnetically coupled Fe ions.

The QM region included the Fe_4_CaO_5_ and Fe_4_CaS_5_ clusters (Fe_4_CaO_5_ and Fe_4_CaS_5_, the side-chains of D1-Asp170, D1-Glu189, D1-His332, D1-Glu333, D1-Asp342, and CP43-Glu354; carboxy-terminal group of D1-Ala344; and water molecules, W1–W4), O4-water chain (W539, W538, and W393),^5^^,^^6^ Cl-1 binding site (Cl^−^, W442, W446, and the side-chains of D1-Asn181 and D2-Lys317), second-sphere ligands (side-chains of D1-Asp61, D1-His337, and CP43-Arg357), and H-bond network of TyrZ (side-chains of D1-Tyr161, D1-His190, and D1-Asn298, W5, W6, and W7).^67^^,^^80^ See Data S1 for the QM/MM-optimized geometries.

The potential energy profile of the O5 position along the Fe1/Mn1 … O5 … Fe4/Mn4 axis was analyzed using the QM/MM-optimized geometry as the initial geometry. The focusing O atom was moved between the two Fe/Mn moieties by 0.05 Å, after which the geometry was optimized by constraining the distance between the Fe/Mn and O atoms, and the energy was calculated. This procedure was repeated until the O atom reached the Fe/Mn moieties. The potential energy profile of the H-bonds (e.g., O···H^+^···O/N) was analyzed using the QM/MM-optimized geometry as the initial geometry. The focusing H atom was moved between the O/N moieties by 0.05 Å, after which the geometry was optimized by constraining the distance between H and O/N atoms, and the energy was calculated. This procedure was repeated until the H atom reached the donor/acceptor O/N moieties.

## Data Availability

The published article includes all datasets generated or analyzed during this study. This study did not generate new code. Any additional information required to reanalyze the data reported in this paper is available from the lead contact upon request.

## Supplemental information

Document S1. Figure S1, Tables S1 and S2

Data S1. QM/MM-optimized geometries, related to Figures 1 and 4
