# Supplementary material for: Structural and energetic insights into Mn-to-Fe substitution in the oxygen-evolving complex
Source: iScience. 2023 Jul 8;26(8):107352. doi: 10.1016/j.isci.2023.107352 (PMC10382916; doi:10.1016/j.isci.2023.107352)
Supplement: Document S1. Figure S1, Tables S1 and S2 [file mmc1.pdf]

iScience, Volume *26*

Supplemental information

Structural and energetic insights into Mn-to-Fe substitution in the oxygen-evolving complex

Masahiro Saito, Keisuke Saito, and Hiroshi Ishikita

Supplemental Information

Structural and Energetic Insights into Mn-to-Fe Substitution in the Oxygen-Evolving Complex

Masahiro Saito [1], Keisuke Saito [1,2], and Hiroshi Ishikita [1,2]*

1) Department of Applied Chemistry, The University of Tokyo, 7-3-1 Hongo, Bunkyo-ku, Tokyo 113-8654, Japan
2) Research Center for Advanced Science and Technology, The University of Tokyo, 4-6-1 Komaba, Meguro-ku, Tokyo 153-8904, Japan

CORRESPONDING AUTHOR: Ishikita, Research Center for Advanced Science and Technology, The University of Tokyo, 4-6-1 Komaba, Meguro-ku, Tokyo 153-8904, Japan, Tel. +81-3-5452-5056, Fax. +81-3-5452-5083, **E-mail:** hiro@appchem.t.u-tokyo.ac.jp

Contents

3 pages

2 tables

Table S1. Bond distances in Fe_4CaO_5, Fe_4CaS_5, and Mn_4CaO_5 clusters in S_2 (Å). Related to Figures 1 and 4.

Fe_4CaO_5		Fe_4CaS_5		Mn_4CaO_5	
Fe1-O1	1.82	Fe1-S1	2.21	Mn1-O1	1.83
Fe1-O3	2.00	Fe1-S3	2.32	Mn1-O3	1.95
Fe1-O5	1.89	Fe1-S5	3.72	Mn1-O5	3.07
Fe2-O1	1.87	Fe2-S1	2.39	Mn2-O1	1.82
Fe2-O2	2.09	Fe2-S2	2.30	Mn2-O2	1.83
Fe2-O3	2.02	Fe2-S3	2.54	Mn2-O3	1.89
Fe3-O2	2.08	Fe3-S2	2.37	Mn3-O2	1.80
Fe3-O3	2.12	Fe3-S3	2.39	Mn3-O3	1.93
Fe3-O4	1.86	Fe3-S4	3.49	Mn3-O4	1.83
Fe3-O5	2.07	Fe3-S5	2.63	Mn3-O5	1.87
Fe4-O4	1.96	Fe4-S4	2.23	Mn4-O4	1.78
Fe4-O5	2.24	Fe4-S5	2.22	Mn4-O5	1.82
Ca-O1	2.36	Ca-S1	2.77	Ca-O1	2.40
Ca-O2	2.40	Ca-S2	3.06	Ca-O2	2.62
Ca-O5	2.49	Ca-S5	2.99	Ca-O5	2.59
Fe1-Fe2	2.86	Fe1-Fe2	3.19	Mn1-Mn2	2.77
Fe2-Fe3	3.08	Fe2-Fe3	3.17	Mn2-Mn3	2.75
Fe3-Fe4	2.77	Fe3-Fe4	3.25	Mn3-Mn4	2.73
Fe4-Fe1	4.10	Fe4-Fe1	5.88	Mn4-Mn1	4.86

Table S2. Spin density of each metal site of the Fe_4CaS_5 cluster in S_2. Related to Table 1.

	**Fe_4CaS_5**
Fe1	3.8
Fe2	3.9
Fe3	3.7
Fe4	3.8
S1	0.6
S2	0.6
S3	0.8
S4	0.5
S5	0.6
Ca	0.0

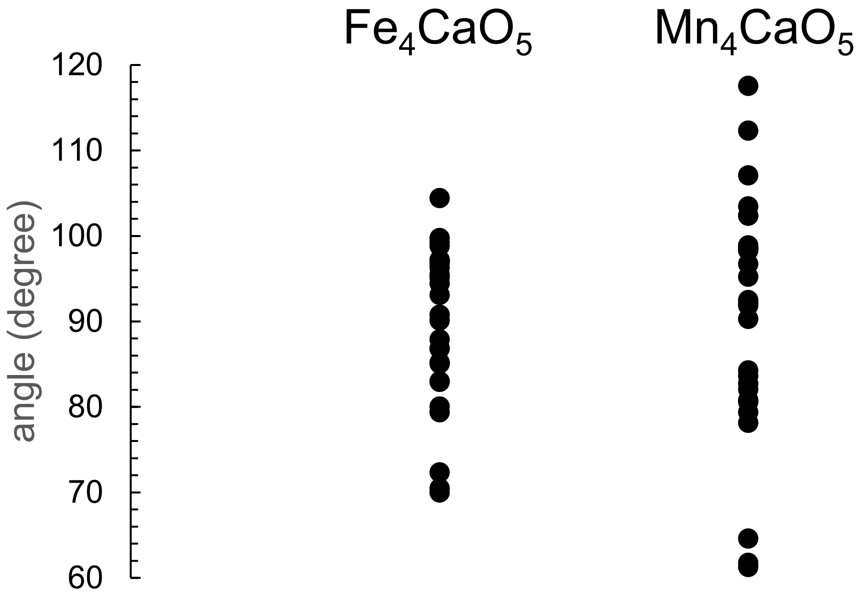

Figure S1. Distribution of angles in the Fe_4CaO_5 and Mn_4CaO_5 clusters. Related to Figures 1 and 4.
